# Genome Sequence and Analysis of *Buzura suppressaria* Nucleopolyhedrovirus: A Group II *Alphabaculovirus*

**DOI:** 10.1371/journal.pone.0086450

**Published:** 2014-01-24

**Authors:** Zheng Zhu, Feifei Yin, Xiaoping Liu, Dianhai Hou, Jun Wang, Lei Zhang, Basil Arif, Hualin Wang, Fei Deng, Zhihong Hu

**Affiliations:** 1 State Key Laboratory of Virology and China Center for Virus Culture Collection, Wuhan Institute of Virology, Chinese Academy of Sciences, Wuhan, China; 2 Canadian Forest Service, Great Lakes Forestry Centre, Sault Ste Marie, Ontario, Canada; Wuhan Bioengineering Institute, China

## Abstract

The genome of Buzura suppressaria nucleopolyhedrovirus (BusuNPV) was sequenced by 454 pyrosequencing technology. The size of the genome is 120,420 bp with 36.8% G+C content. It contains 127 hypothetical open reading frames (ORFs) covering 90.7% of the genome and includes the 37 conserved baculovirus core genes, 84 genes found in other baculoviruses, and 6 unique ORFs. No typical baculoviral homologous repeats (*hrs*) were present but the genome contained a region of repeated sequences. Gene Parity Plots revealed a 28.8 kb region conserved among the alpha- and beta-baculoviruses. Overall comparisons of BusuNPV to other baculoviruses point to a distinct species in group II *Alphabaculovirus*.

## Introduction

The *Baculovirdae* is an insect-specific family of viruses with double stranded circular DNA genomes of 80 kb –180 kb. Among the so far sequenced baculoviruses, Xestia c-nigrum granulovirus (XecnGV) has the largest genome (178,733 bp) with the smallest in the Neodiprion lecontei nucleopolyhedrovirus (NeleNPV, 81,755 bp) [1], [2]. With the exception of members of *Gammabaculovirus*, two distinct progeny phenotypes are produced, the budded virus (BV) that disseminates systemically and the occlusion derived virus (ODV) required for oral infectivity [3]. The occlusion bodies (OBs) afford the embedded virions a certain amount of protection against environmental inactivating conditions such as UV lights and rainwater. The number of predicted ORFs in a single baculovirus range from 89 (NeleNPV) to 183 (Pseudaletia unipuncta GV, PsunGV) [2]. Among all the baculovirus predicted ORFs, 37 have been identified as core genes that exist in all sequenced baculoviruses and are essential for the viral life cycle [4], [5].

The family *Baculoviridae* is classified into 4 genera: *Alphabaculovirus* (NPVs isolated from Lepidoptera); *Betabaculovirus* (GVs isolated from Lepidoptera); *Gammabaculovirus* (NPVs isolated from Hymenoptera) and *Deltabaculovirus* (NPVs isolated from Diptera) [6], [7]. The *Alphabaculovirus* are further clustered into groups I and II based on phylogenetic analyses and the presence or absence of the *gp64* gene. Only group I contains *gp64* gene while group II has a gene encoding fusion protein (F) [8]–[11].

*Buzura suppressaria* is a pest insect of tea, tung oil, citrus and metasequoia plants. The Buzura suppressaria NPV (BusuNPV) was first isolated from dead larva of *B. suppressaria* and subsequently used as an insecticide against this pest [12], [13]. The virus is a single nucleocapsid NPV with a genome size of approximately 120 kb. So far, only a few of the BusuNPV genes have been identified, including those encoding polyhedrin [12], [14], ecdysteroid UDP-glucosyltransferase (egt) [15], polyhedron envelope protein gene (*pep*), the conotoxin-like protein gene (*ctl*), the inhibitor of apoptosis (*iap*), superoxide dismutase (*sod*) [16], and P10 [17]. A physical map of viral DNA was determined [12] and about 43.5 kb dispersed regions of the genome have been sequenced showing a distinct gene arrangement of BusuNPV [13]. In this manuscript we report the complete genome of BusuNPV. Sequence analysis showed that BusuNPV is a group II *Alphabaculovirus* with a genome distinct from other so far sequenced baculoviruses.

## Results and Discussion

### Sequencing and Genome Characteristics

The genome of BusuNPV was sequenced using the Roche 454 GS FLX system with shotgun strategy. A total of 97,246 reads were obtained with the average length of 340 bp. The BusuNPV genome was assembled by Roche GS De Novo assembler software and assisted by the published restriction maps [13]; the genome was covered 217 times.

**Figure 1:**
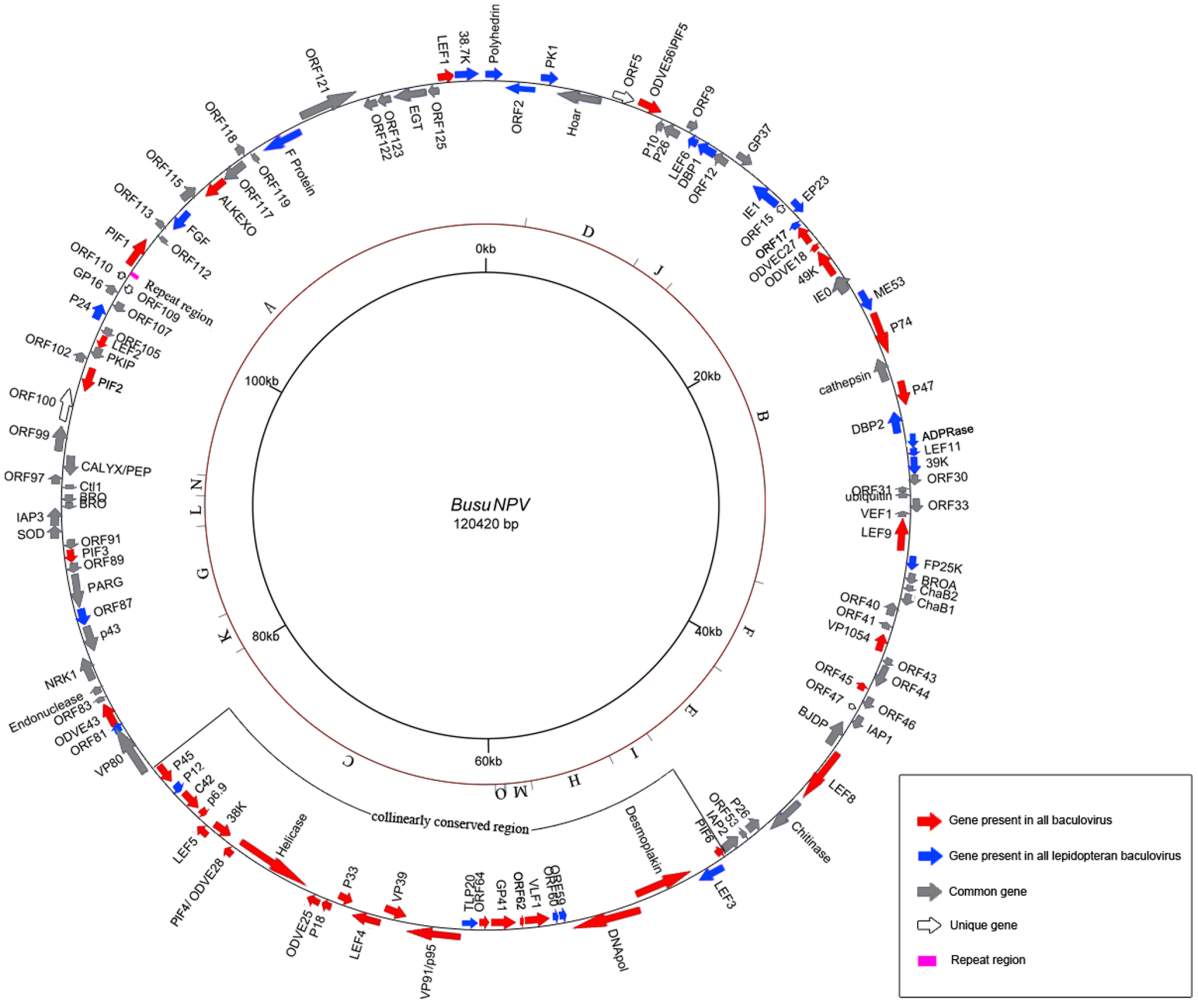
Genome map of BusuNPV. ORFs are indicated by arrows with a displayed name. Arrows also signify transcription directions. Red arrows represent core genes, blue represent genes present in all lepidopteron baculoviruses, gray represent baculoviral common genes and open arrowers represent unique genes of BusuNPV. The pink square represent a repeat structure. The inner circle indicates genome scale position by 20*Hin*dIII restriction map is shown in the middle dark red circle. A region collinearly conserved in alpha- and betabaculoviruses is also shown.

The size of the BusuNPV genome is 120,420 bp with a G+C content of 36.8% (Table S1) and 127 hypothetical ORFs of more than 150 bp. The *polyhedrin* gene was defined as the first ORF and the A of its initiation codon as the first nucleotide (nt) of the genome. So far, 78 baculoviral genomes have been completely sequenced including BusuNPV (Table S1). BusuNPV contains the 37 core genes conserved in all baculoviruses (shown as red in Fig. 1) and 25 other genes that are present in all sequenced lepidopteran baculovirus (shown as blue in Fig. 1). The genome also contains 59 additional genes commonly found in a variety of baculoviruses (shown as grey in Fig. 1) and also has 6 unique genes (shown as open arrows, Fig. 1). A restriction map of *Hin*dIII is presented in Fig. 1, which corroborates the previous physical map [13]. A region appears to be conserved in alpha- and beta-baculoviruses (see below) is also presented in this figure.

### Classification of BusuNPV

**Figure 2:**
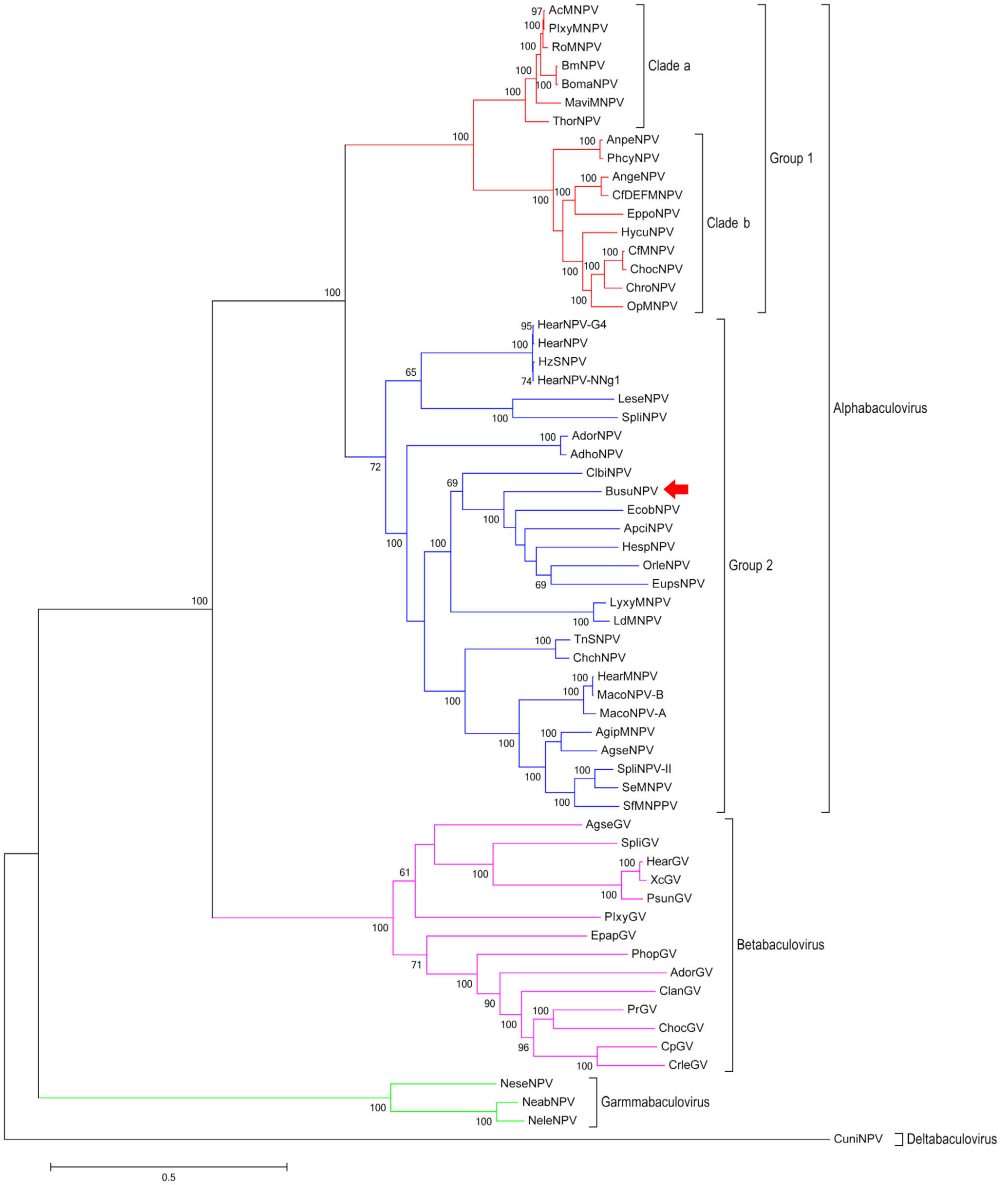
Phylogenetic tree using 37 core proteins of 62 sequenced baculoviruses based on Maximum Likelihood method. It tested by Bootstrap method with a value of 1000. The bootstrap values greater than 50% are showed in front of every nodes. Arrow points to BusuNPV.

Phyogenetic analysis on the 37 core proteins from the 62 representing baculoviruses placed BusuNPV in group II of the genus *Alphabaculovirus* (Fig. 2), which is consistent with the previous reports [13], [16]. It formed a subclade with other six NPVs including Ectropis obliqua NPV (EcobNPV), Apocheima cinerarium NPV (ApciNPV), Euproctis pseudoconspersa NPV (EupsNPV), Hemileuca sp. NPV (HespNPV), Clanis bilineata NPV (ClbiNPV) and Orgyia leucostigma NPV (OrleNPV) [18], [19].

### Comparison to other Baculoviruses

The nucleotide identities between the ORFs of BusuNPV and other representative baculoviruses are shown in Table S2. The overall genomic nucleic acid identity to EcobNPV, EupsNPV, OrleNPV, HespNPV, ClbiNPV and ApciNPV was about 27.2%, 27.0%, 26.7% 22.0%, 24.2% and 27.4%, respectively. The observed low identities imply that BusuNPV is evolutionarily quite divergent from the fully sequenced baculoviruses.

**Figure 3:**
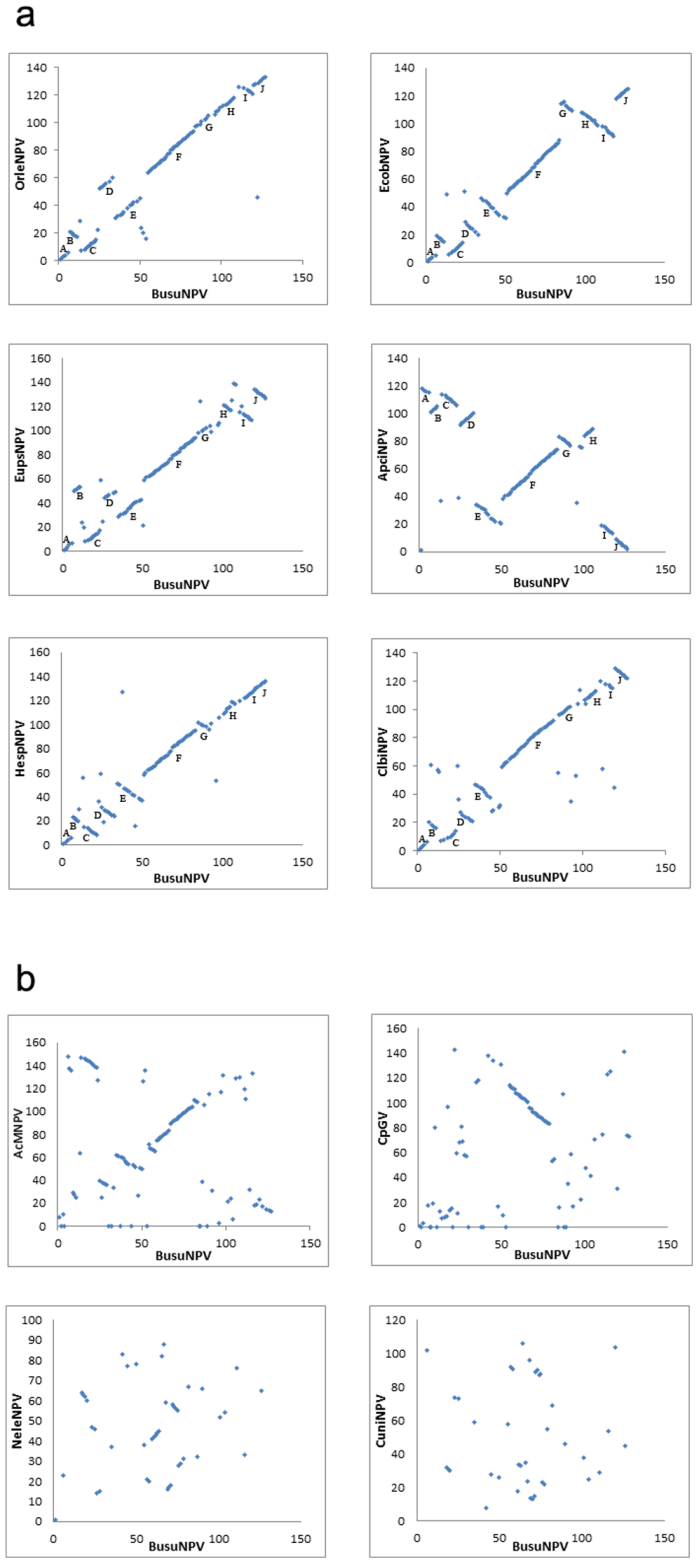
Gene-parity plot analysis. **a**. Gene-parity plots of BusuNPV with OrleNPV, EupsNPV, ApciNPV, HespNPV, ClbiNPV and EcboNPV based on ORF order. The gene cluster marked by alphabet sorted by their order in BusuNPV. **b**. Gene-parity plot of BusuNPV with AcMNPV, HearNPV G4, CpGV, NeleNPV and CuniNPV based on ORF order.

Gene-parity plots of BusuNPV against the other 6 viruses in the same subclade demonstrated colinearity with some inversions over the whole genome (Fig. 3a). Some colinearity was also found with representatives of group I alphabaculoviruses and betabaculoviruses, but almost no colinearity with those from gamma- and deltabaculoviruses (Fig. 3b). Interestingly, a 28.8 kb region from Busu55 to Busu79 is almost totally collinearly conserved in alpha- and betabaculoviruses (Table 1, Fig. 1). This region contains 25 ORFs in BusuNPV, 20 of which are conserved in all baculoviruses (Table 1, Fig. 1). It is likely that this region existed in the common ancestor of alpha- and betabaculoviruses.

**Table 1:** Collinearly conserved region in alpha- and betabaculoviruses#.

Gene name	ORF position				
	BusuNPV	AcMNPV	HearNPV	LdMNPV	CpGV
PIF-6*	55	68	64	80	114
LEF-3	56	67	65	81	113
Desmoplakin*	57	66	66	82	112
DNA-pol*	58	65	67	83	111
ORF-59	59	75	69	84	108
ORF-60	60	76	70	85	107
VLF-1*	61	77	71	86	106
P78/83*	62	78	72	87	105
GP41*	63	80	73	88	104
AC81*	64	81	74	89	103
TLP-20$	65	82	75	90	102
VP91/p95*	66	83	76	91	101
VP39*	67	89	78	92	96
LEF-4*	68	90	79	93	95
P33*	69	92	80	94	93
P18*	70	93	81	95	92
ODV-E25*	71	94	82	96	91
Helicase*	72	95	84	97	90
ODV-E28/PIF-4*	73	96	85	98	89
38K*	74	98	86	99	88
LEF-5*	75	99	87	100	87
p6.9*	76	100	88	101	86
C42*	77	101	89	102	85
P12	78	102	90	103	84
P45*	79	103	91	104	83

# The collinearity was shown by the ORFs orders in BusuNPV, AcMNPV, HearNPV G4, LdMNPV and CpGV. Conserved ORF of all baculovirus are marked by ‘*’.

$ TLP means Telokin-like protein.

### Repeat Structures

Homologous repeated sequences (*hrs*) were supposed to be characteristic for many baculovirus genomes. The *hrs* are repeat regions with palindrome structure interspersed in the genome. *Hr*s consist of similar repeat sequence with varying length in a genome, but the *hr* sequence vary widely in different baculoviruses [20]. *Hrs* were suggested to be origins of DNA replication in baculovirus [21], [22], however, a contrasting study showed deletion individual *hr* had no effect on the replication of AcMNPV [23]. Other studies attributed an enhancer function to *hrs*. They appear to bind to *ie1* in AcMNPV and promote the transactivation level of IE1 [24]–[26]. *Hrs* are absent from the *Busu*NPV genome.

**Figure 4:**
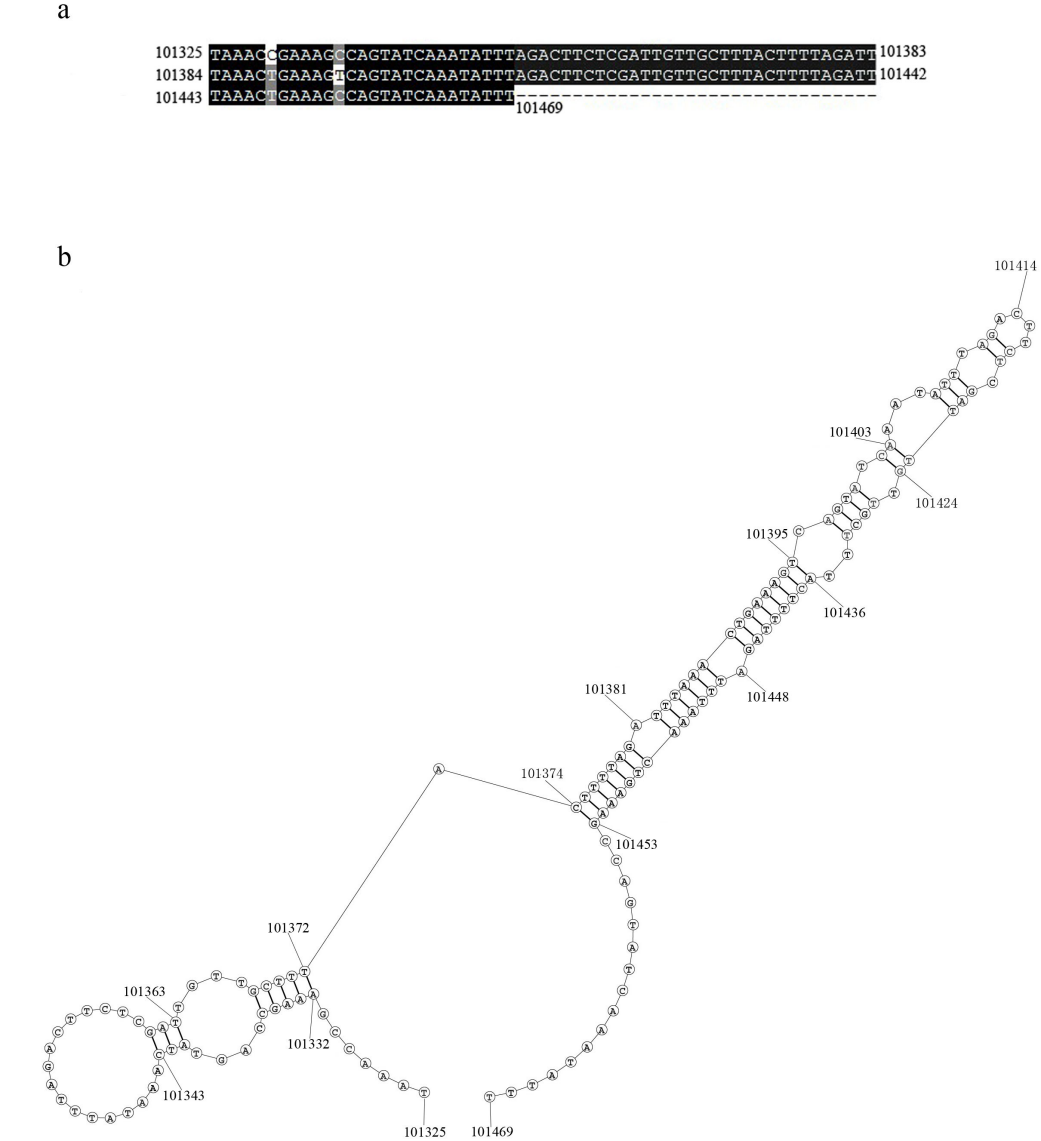
Repeat structure of BusuNPV. **a.** Sequence comparing of repeat regions. Blank background shows same bases between 3 compared regions, gray indicates same bases only in 2 regions. **b.** Predicted secondary structure of overlap repeat region. Numbers on both sides of the chains are the base position in the genome.

A *non-hr* origin was also suggested to initiate replication which contains palindromic and repetitive sequences in a complex organization [21], [27]. A repeat sequence was detected from nt 101325 to 101469 in the BusuNPV genome and contained two complete repeats and a truncated repeat. The repeat is 59 nt (Fig. 4a), high in A+T content (71.7%) and probably forms a hairpin structure (Fig. 4b). Whether this is a functional non-hr origin for BusuNPV needs further analysis.

### Replication Genes

Although the mechanism of baculovirus genome replication is not totally clear, several viral genes have been identified as important for DNA replication [28]. BusuNPV encodes genes essential for replication including DNA polymerase (Busu58), DNA helicase (Busu72), late expression factor-1 (*lef-1*, Busu126), *lef-2* (Busu104) and *lef-3* (Busu56). Other genes related to DNA replication include very late factor-1 (*vlf-1*, Busu61), DNA binding proteins-1,2 (*dbp-1*, Busu11 and *dbp*-2, Busu26), *lef-11* (Busu28), alkaline exonuclease (*alk-exo,* busu116) and *me-53* (Busu22) [29] have also identified in BusuNPV.

### Transcription Genes

Like all other baculovirus, BusuNPV encodes all four subunits of RNA polymerase [30], *lef*-4 (Busu68), *lef*-8 (Busu50), *lef*-9 (Busu35) and P47 (*Busu*25). The *lef-5* (*Busu*75) and *vlf-1* (*Busu*61) are two other core genes involved in transcription. In addition, four non-core transcription related gene: *39k/pp31* (Busu29), *lef-6* (Busu10), *lef-11* (Busu28), Protein kinase-1 (*pk-1,* Busu3) are present in BusuNPV. The early transcription genes found in BusuNPV are Immediate early gene (*ie-1,* Busu14) and *ie-0* (Busu21) [31], [32].

### Structural Genes

BusuNPV contains all structural core genes identified in other baculoviruses. In the alphabaculoviruses, *p6.9* (Busu76) encodes a nucleocapsid protein and participates in DNA condensation. VLF-1 (Busu61) is a structural protein in both ODV and BV required for very late genes expression and is essential for nucleocapsid production [33], [34]. Other core genes related to the viral nucleocapsid include *38K* (Busu74), *49k* (Busu20), *odv-ec27* (Busu18), *odv-e43* (Busu82), *odv-e18* (Busu19), *vp39* (Busu67), *vp91/p95* (Busu66), *vp1054* (Busu42), *desmoplakin* (Busu57), Ac53 (Busu45), *p18* (Busu70) and *gp41* (Busu63). The *p33* (Busu69) encodes a type of a sulfhydryl oxidase in baculoviruses [35]. Proteins encoded by *c42* (Busu77) and pp78/83 (Busu2) participate in nuclear actin polymerization [36]. Busu62 encodes a protein similar to Ha72, which be verified essential for ODV occlusion and BV production [37].

Other non-core structural proteins encompass the F protein (Busu120), which is essential for virus entry and budding and VP80 (Busu80), which is involved in nucleocapsid packaging and trafficking [38]. Busu98 is a homologue of Calyx/PEP and is the major protein of polyhedron envelope that enhances the stability of OBs [39], [40]. Busu7 encodes P10 [17] and is involved in the process of OB envelopment and nuclear lysis at the late stages of infection [41].

### Oral Infectivity Factors

So far 7 conserved genes were identified to be essential for oral infectivity of baculovirus including *p74* (Busu23), per os infectivity factors-1 (*pif-1,* Busu111), *pif-2* (Busu101), *pif-3* (Busu90), *pif-4* (Busu73), *pif-5/odv-e56* (Busu6) and *pif-6*(Busu55) [42], [43].

*Busu34* is a homologue of the gene encoding viral enhancing factor (VEF) that dissolves the peritrophic membrane (PM) of the midgut [44]. A study in LdMNPV found it helps ODV envelopes [45].

### Auxiliary Genes

Ubiquitin is encoded by most baculoviruses as well as BusuNPV. Like most alphabaculoviruses and some betabaculoviruses, BusuNPV also encodes *cathepsin* (Busu24) and *chitinase* (Busu51), both are involved in liquefaction of insect and OB release [46], [47]. A fibroblast growth factor (FGF, Busu114) aids virus dissemination through the tracheal system [48], [49]. The *egt* gene which prevents larvae molting and pupation [50], [51] was found in BusuNPV (Busu124) [15] and the baculovirus with deficiency *egt* gene kill the infected larvae faster than wild type stains [52], [53]. BusuNPV also contains a *sod* (Busu92) and three *iap* genes (*iap-1*, Busu48; *iap-2*, Busu54; and *iap-3,* Busu93). Three Baculovirus repeated orf (*bro*) genes have also been found. The absence or duplication of these genes is common in baculovirus, although between stains with closer affinity [54]. A study on BmNPV showed that mutant bro-d or double mutant bro-a and bro-c could not be isolated, it suggested bro takes essential functions in BmNPV [55]. Another study indicated *bro* genes encode a protein with DNA binding activity, especially to single stranded DNA [56]. BusuNPV encodes poly (ADP-ribose) glycohydrolase (*parg*, Busu88), which is conserved in group II alphabaculoviruses with a function of poly (ADP-ribose) catabolism [29]. A study in HearNPV G4 showed it affects oral infectivity of OBs [57].

### Unique Genes

Six unique ORFs (Busu5, Busu15, Busu47, Busu100, Busu 109 and Busu110) with no homology to other baculovirus ORFs were identified and potentially encode functional proteins.

The Busu100 encodes a 532 aa protein with low homology to tryptophan repeat gene family in entomopoxvirus (minimum E value = 0.012). Busu109 encodes a 155 aa protein sharing a very low homology to 5-methyltetrahydropteroyltriglutamate–homocysteine methyltransferase in some bacteria (minimum E value = 2.1).

In summary, the genome sequence revealed BusuNPV is a distinct species in group II *Alphabaculovirus*. Phylogenetically, it is most closely related to EcobNPV, EupsNPV, OrleNPV and ApciNPV. It does not contain typical baculovirus *hrs*, but contain a new repeat structure, the function of which needs to be further characterized. A 28.8 kb conserved region was identified among alpha- and betabaculoviruses.

## Materials and Methods

### Viral DNA Extraction

BusuNPV was propagated in *B. suppressaria* larvae and OBs were purified by differential centrifugation [12]. DNA was extracted as described previously [16].

### Sequencing and Bioinformatic Analysis

The genome was sequenced with the Roche 454 GS FLX system by using shotgun strategy. The reads were assembled with Roche GS De Novo assembler software. Contigs assembly was assisted by previously generated restriction maps [13]. A few regions that were not assembled into the contigs were further amplified by PCR, cloned and sequenced. The genome sequence data was uploaded to GenBank (GenBank accession number: KF611977).

Hypothetical ORFs were predicted by softberry FGENESV program (http://www.softberry.com/berry.phtml) [58] to contain the standard ATG start, and a stop codon and potentially encode at least 50 amino acids. Gene-parity plot analysis [13] was performed using Microsoft Office Excel to draw scatter diagram with using BusuNPV ORFs number as the X-axis and other baculovirus ORFs as the Y-axis. Gene annotation and comparisons were done with NCBI protein-protein BLAST algorithm (http://blast.ncbi.nlm.nih.gov/Blast.cgi). Repeat structures were detected by BLAST alignment of two sequences (http://blast.ncbi.nlm.nih.gov/Blast.cgi). The identity among homologous genes was done with MegAlign software using clustalW with default parameters. Regulatory regions and promoter motifs were identified as described previously [29].

Restriction sites were predicted by MapDraw software. Genome map framework drawn with genomeVX [59].

### Phylogenetic Analysis

The Phylogenetic analysis was based on amino acid sequences of 37 core genes form BusuNPV and the other 61 baculoviruses listed in NCBI genome database (Table S1). All the sequences were linked by a stationary order and multiple alignments using clusterW method with MEGA5 by using default settings. A phylogenetic tree was constructed by MEGA5 using Maximum Likelihood method based on the JTT matrix-based model [60], [61]. Phylogeny tested by Bootstrap method with a value of 1000 [62].

### Prediction of Secondary Structure

Secondary structure was drawn by Predict a Secondary Structure online server (http://rna.urmc.rochester.edu/RNAstructureWeb/Servers/Predict1/Predict1.html) with default setting of DNA Nucleic Acid Type [63].

## Supporting Information

Table S1 Basic informationof all sequenced baculovirus genome in Genbank (October, 2013). NP means no published. Genomes used to build phylogeny tree marked by ‘ *’.
(DOCX)

Table S2 The ORF positions in the genomeof BusuNPV. E or L means early or late promoter motif and ORF directionrepresented by+ or –.* stands for stain HearNPV G4. a, position of granulin in CpGV genome. b, BJDP stands for DnaJ domain protein. c, PKIP stands forProtein kinase interacting.
(DOCX)
