# Supplementary material for: Genome Sequence and Analysis of Buzura suppressaria Nucleopolyhedrovirus: A Group II Alphabaculovirus
Source: PLoS One. 2014 Jan 24;9(1):e86450. doi: 10.1371/journal.pone.0086450 (PMC3901692; doi:10.1371/journal.pone.0086450)
Supplement: Table S1 — Basic informationof all sequenced baculovirus genome in Genbank (October, 2013). NP means no published. Genomes used to build phylogeny tree marked by ‘ *’. (DOCX) [file pone.0086450.s001.docx]

| Genus | Genome  Table s1. Basic information of all sequenced baculovirus genome in GenBank(October,2013) ^#^ | GeneBank accession | G+C  (%) | hrs | bro | Genome  size (bp) | ORFs | Reference |
| --- | --- | --- | --- | --- | --- | --- | --- | --- |
| Alphabaculovirus(GroupI) | *Antheraea pernyi* MNPV* | DQ486030 | 53.4 | 3 | 2 | 126629 | 147 | [[1](#_ENREF_1)] |
|  | *Antheraea pernyi* MNPV L2 | EF207986 | 53.5 | 6 | 2 | 126246 | 145 | [[2](#_ENREF_2)] |
|  | *Anticarsia gemmatalis* MNPV* | DQ813662 | 44.5 | 9 | 8 | 132239 | 152 | [[3](#_ENREF_3)] |
|  | *Autographa californica* MNPV* | L22858 | 40.7 | 9 | 1 | 133894 | 156 | [[4](#_ENREF_4)] |
|  | *Bombyx mori* NPV T3* | L33180 | 40.4 | 7 | 5 | 128413 | 143 | [[5](#_ENREF_5)] |
|  | *Bombyx mori* NPV Cubic | JQ991009 | 40.4 | 7 | 5 | 127465 | 142 | [[6](#_ENREF_6)] |
|  | *Bombyx mori* NPV Zhejiang | JQ991008 | 40.3 | 7 | 4 | 126125 | 134 | [[7](#_ENREF_7)] |
|  | *Bombyx mori* NPV India | JQ991010 | 40.3 | 7 | 4 | 126879 | 133 | [[8](#_ENREF_8)] |
|  | *Bombyx mori* NPVGuangxi | JQ991011 | 40.4 | 7 | 4 | 126843 | 134 | [[7](#_ENREF_7)] |
|  | *Bombyx mandarina* NPV s1* | FJ882854 | 40.2 | 7 | 3 | 126770 | 141 | [[9](#_ENREF_9)] |
|  | *Bombyx mandarina* NPV s2 | JQ071499 | 59.6 | 8 | 5 | 129646 | 135 | [[10](#_ENREF_10)] |
|  | *Choristoneura fumiferana* MNPV* | AF512031 | 50.1 | 5 | 1 | 129593 | 146 | [[11](#_ENREF_11)] |
|  | *Choristoneura fumiferana* DEF MNPV* | AY327402 | 45.8 | 13 | 4 | 131160 | 149 | [[12](#_ENREF_12)] |
|  | *Choristoneura occidentalis* NPV* | KC961303 | 50.1 | 5 | 1 | 128446 | 148 | [[13](#_ENREF_13)] |
|  | *C. rosaceana* NPV* | KC961304 | 48.6 | 3 | 2 | 129052 | 149 | [[13](#_ENREF_13)] |
|  | *Epiphyas postvittana* MNPV* | AY043265 | 40.7 | 5 | 1 | 118584 | 136 | [[14](#_ENREF_14)] |
|  | *Hyphantria cunea* MNPV* | AP009046 | 45.1 | 6 | 5 | 132959 | 148 | [[15](#_ENREF_15)] |
|  | *Maruca vitrata* MNPV* | EF125867 | 38.6 | 5 | 0 | 111953 | 126 | [[16](#_ENREF_16)] |
|  | *Orgyia pseudotsugata* MNPV* | U75930 | 55.1 | 5 | 1 | 131995 | 152 | [[17](#_ENREF_17)] |
|  | *Philosamia cynthia* NPV* | JX404026 | 53.6 | 6 | 2 | 125376 | 138 | [[18](#_ENREF_18)] |
|  | *Plutella xylostella* MNPV* | DQ457003 | 40.7 | 9 | 2 | 134417 | 152 | [[19](#_ENREF_19)] |
|  | *Rachiplusia ou* MNPV* | AY145471 | 39.1 | 9 | 0 | 131526 | 149 | [[20](#_ENREF_20)] |
|  | *Thysanoplusia orichalcea* MNPV * | JX467702 | 37.9 | 6 | 2 | 132978 | 145 | [[21](#_ENREF_21)] |
| Alphabaculovirus(GroupII) | *Adoxophyes honmai* SNPV ADN001* | AP006270 | 35.6 | 4 | 4 | 113220 | 125 | [[22](#_ENREF_22)] |
|  | *Adoxophyes orana* SNPV* | EU591746 | 35.0 | 4 | 3 | 111724 | 121 | [[23](#_ENREF_23)] |
|  | *Agrotis ipsilon* MNPV* | EU839994 | 48.6 | 7 | 5 | 155122 | 163 | [[24](#_ENREF_24)] |
|  | *Agrotis segetum* MNPV* | DQ123841 | 45.7 | 5 | 4 | 147544 | 153 | [[25](#_ENREF_25)] |
|  | *Apocheima cinerarium* SNPV* | FJ914221 | 33.4 | 4 | 1 | 123876 | 117 | NP |
|  | *Burura suppressaria* SNPV* | KF611977 | 36.8 | 0 | 3 | 120420 | 127 | - |
|  | *Chrysodeixis chalcites* SNPV* | AY864330 | 39.1 | 0 | 4 | 149622 | 151 | [[26](#_ENREF_26)] |
|  | *Clanis bilineata* SNPV DZ1* | DQ504428 | 37.7 | 0 | 3 | 135454 | 129 | [[27](#_ENREF_27)] |
|  | *Ectropis obliqua* SNPV A1* | DQ837165 | 37.6 | 3 | 2 | 131204 | 126 | [[28](#_ENREF_28)] |
|  | *Euproctis pseudoconspersa* MNPV* | FJ227128 | 40.4 | 4 | 2 | 141291 | 139 | [[29](#_ENREF_29)] |
|  | *Helicoverpa armigera* SNPV G4* | AF271059 | 39.1 | 5 | 3 | 131403 | 135 | [[30](#_ENREF_30)] |
|  | *Helicoverpa armigera* SNPV C1* | AF303045 | 38.9 | 5 | 3 | 130759 | 137 | [[31](#_ENREF_31)] |
|  | *Helicoverpa armigera* SNPV NNg1* | AP010907 | 39.1 | 5 | 4 | 132425 | 143 | [[32](#_ENREF_32)] |
|  | *Helicoverpa armigera* MNPV* | EU730893 | 39.7 | 4 | 6 | 154196 | 162 | [[33](#_ENREF_33)] |
|  | *Helicoverpa armigera* NPV Australia | JN584482 | 39 | 5 | 2 | 130992 | 134 | NP |
|  | *Helicoverpa zea* SNPV* | AF334030 | 39.1 | 5 | 2 | 130869 | 139 | [[34](#_ENREF_34)] |
|  | *Hemileuca sp.* NPV* | KF158713 | 38.1 | 3 | 2 | 140633 | 137 | [[35](#_ENREF_35)] |
|  | *Leucania separata* MNPV AH1* | AY394490 | 48.6 | 8 | 10 | 168041 | 169 | [[36](#_ENREF_36)] |
|  | *Lymantria dispar* MNPV* | AF081810 | 57.5 | 13 | 16 | 161046 | 164 | [[37](#_ENREF_37)] |
|  | *Lymantria xylina* MNPV* | GQ202541 | 53.4 | 13 | 14 | 156344 | 157 | [[38](#_ENREF_38)] |
|  | *Mamestra brassicae* MNPV K1 | JQ798165 | 39.8 | 4 | 6 | 152710 | 159 | [[39](#_ENREF_39)] |

| Genus | Genome | Genebank accession | G+C  (%) | hrs | bro | Genome  size (bp) | ORFs | Reference |
| --- | --- | --- | --- | --- | --- | --- | --- | --- |
|  | *Mamestra brassicae* MNPV CHb1 | JX138237 | 40.1 | 4 | 6 | 154432 | 163 | NP |
| Alphabaculovirus(GroupII) | *Mamestra configurata* NPV A 90/2* | U59461 | 41.7 | 4 | 8 | 155060 | 169 | [[40](#_ENREF_40)] |
|  | *Mamestra configurata* NPV A 90/4 | AF539999 | 41.7 | 4 | 7 | 153656 | 168 | [[41](#_ENREF_41)] |
|  | *Mamestra configurata* NPV 96 B* | AY126275 | 40.4 | 4 | 7 | 158482 | 168 | [[42](#_ENREF_42)] |
|  | *Orgyia leucostigma* NPV* | EU309041 | 39.9 | 3 | 5 | 156179 | 135 | [[43](#_ENREF_43)] |
|  | *Spodoptera exigua* MNPV* | AF169823 | 43.8 | 6 | 0 | 135611 | 139 | [[44](#_ENREF_44)] |
|  | *Spodoptera frugiperda* MNPV 3AP2* | EF035042 | 40.2 | 8 | 1 | 131331 | 143 | [[45](#_ENREF_45)] |
|  | *Spodoptera frugiperda* MNPV 19 | EU258200 | 40.3 | 8 | 1 | 132565 | 141 | [[46](#_ENREF_46)] |
|  | *Spodoptera frugiperda* MNPV Nicaraguan | HM595733 | 40.3 | 8 | 1 | 132954 | 143 | [[47](#_ENREF_47)] |
|  | *Spodoptera frugiperda* MNPV DEF | JF899325 | 40.3 | 8 | 1 | 128034 | 143 | [[48](#_ENREF_48)] |
|  | *Spodoptera litura* MNPV G2* | AF325155 | 42.7 | 17 | 2 | 139342 | 141 | [[49](#_ENREF_49)] |
|  | *Spodoptera litura* MNPV II* | EU780426 | 45.0 | 7 | 2 | 148634 | 147 | NP |
|  | *Spodoptera litura* MNPV AN1956 | JX454574 | 44.7 | 15 | 1 | 137998 | 132 | [[50](#_ENREF_50)] |
|  | *Trichoplusia ni* SNPV* | DQ017380 | 39.0 | 0 | 2 | 134394 | 145 | [[51](#_ENREF_51)] |
| Betabaculovirus | *Adoxophyes orana* GV* | AF547984 | 34.5 | 0 | 0 | 99657 | 119 | [[52](#_ENREF_52)] |
|  | *Agrotis segetum* GV* | AY522332 | 37.3 | 0 | 0 | 131680 | 132 | NP |
|  | *Choristoneura occidentalis* GV* | DQ333351 | 32.7 | 5 | 0 | 104710 | 116 | [[53](#_ENREF_53)] |
|  | *Clostera anachoreta* GV* | HQ116624 | 44.4 | 4 | 0 | 101487 | 123 | [[54](#_ENREF_54)] |
|  | *Clostera anastomosis* GV CaLGV-Henan | KC179784 | 46.7 | 1 | 0 | 101818 | 123 | [[55](#_ENREF_55)] |
|  | *Cryptophlebia leucotreta* GV CV3* | AY229987 | 32.4 | 3 | 0 | 110907 | 128 | [[56](#_ENREF_56)] |
|  | *Cydia pomonella* GV* | U53466 | 45.3 | 0 | 1 | 123500 | 143 | [[57](#_ENREF_57)] |
|  | *Epinotia aporema* GV* | JN408834 | 41.5 | 16 | 0 | 119082 | 132 | [[58](#_ENREF_58)] |
|  | *Helicoverpa armigera* GV* | EU255577 | 40.8 | 9 | 10 | 169794 | 179 | [[59](#_ENREF_59)] |
|  | *Phthorimaea operculella* GV* | AF499596 | 35.7 | 12 | 1 | 119217 | 130 | [[60](#_ENREF_60)] |
|  | *Pieris rapae* GV* | GQ884143 | 33.2 | 0 | 0 | 108592 | 120 | [[61](#_ENREF_61)] |
|  | *Pieris rapae* granulovirus E3 | GU111736 | 33.2 | 0 | 0 | 108476 | 125 | NP |
|  | *Plutella xylostella* GV K1* | AF270937 | 40.7 | 4 | 0 | 100999 | 120 | [[62](#_ENREF_62)] |
|  | *Pseudaletia unipuncta* GV* | EU678671 | 39.8 | 9 | 7 | 176677 | 183 | NP |
|  | *Spodoptera litura GV K1** | DQ288858 | 38.8 | 0 | 6 | 124121 | 136 | [[63](#_ENREF_63)] |
|  | *Xestia c-nigrum* GV* | AF162221 | 40.7 | 9 | 7 | 178733 | 181 | [[64](#_ENREF_64)] |
| Gamma- baculovirus | *Neodiprion abietis* NPV* | DQ317692 | 33.4 | 5 | 0 | 84264 | 93 | [[65](#_ENREF_65)] |
|  | *Neodiprion lecontei* NPV* | AY349019 | 33.4 | 0 | 0 | 81755 | 89 | [[66](#_ENREF_66)] |
|  | *Neodiprion sertifer* NPV* | AY430810 | 33.8 | 6 | 0 | 86462 | 90 | [[67](#_ENREF_67)] |
| Delta-  baculovirus | *Culex nigripalpus* NPV* | AF403738 | 50.9 | 4 | 6 | 108252 | 109 | [[68](#_ENREF_68)] |

#Table S1. Basic information of all sequenced baculovirus genome in Genbank (October, 2013). NP means no published. Genomes used to build phylogeny tree marked by ‘ *’.

Reference

1. Nie ZM, Zhang ZF, Wang D, He PA, Jiang CY, et al. (2007) Complete sequence and organization of Antheraea pernyi nucleopolyhedrovirus, a dr-rich baculovirus. BMC Genomics 8: 248.

2. Fan Q, Li S, Wang L, Zhang B, Ye B, et al. (2007) The genome sequence of the multinucleocapsid nucleopolyhedrovirus of the Chinese oak silkworm Antheraea pernyi. Virology 366: 304-315.

3. Oliveira JV, Wolff JL, Garcia-Maruniak A, Ribeiro BM, de Castro ME, et al. (2006) Genome of the most widely used viral biopesticide: Anticarsia gemmatalis multiple nucleopolyhedrovirus. J Gen Virol 87: 3233-3250.

4. Ayres MD, Howard SC, Kuzio J, Lopez-Ferber M, Possee RD (1994) The complete DNA sequence of Autographa californica nuclear polyhedrosis virus. Virology 202: 586-605.

5. Gomi S, Majima K, Maeda S (1999) Sequence analysis of the genome of Bombyx mori nucleopolyhedrovirus. J Gen Virol 80 ( Pt 5): 1323-1337.

6. Cheng RL, Xu YP, Zhang CX (2012) Genome sequence of a Bombyx mori nucleopolyhedrovirus strain with cubic occlusion bodies. J Virol 86: 10245.

7. Xu YP, Cheng RL, Xi Y, Zhang CX (2013) Genomic diversity of Bombyx mori nucleopolyhedrovirus strains. Genomics 102: 63-71.

8. Fan HW, Zhang XC, Xu YP, Cheng XW, Zhang CX (2012) Genome of a Bombyx mori nucleopolyhedrovirus strain isolated from India. J Virol 86: 11941.

9. Xu YP, Ye ZP, Niu CY, Bao YY, Wang WB, et al. (2010) Comparative analysis of the genomes of Bombyx mandarina and Bombyx mori nucleopolyhedroviruses. J Microbiol 48: 102-110.

10. Xu YP, Gu LZ, Lou YH, Cheng RL, Xu HJ, et al. (2012) A baculovirus isolated from wild silkworm encompasses the host ranges of Bombyx mori nucleopolyhedrosis virus and Autographa californica multiple nucleopolyhedrovirus in cultured cells. J Gen Virol 93: 2480-2489.

11. de Jong JG, Lauzon HA, Dominy C, Poloumienko A, Carstens EB, et al. (2005) Analysis of the Choristoneura fumiferana nucleopolyhedrovirus genome. J Gen Virol 86: 929-943.

12. Lauzon HA, Jamieson PB, Krell PJ, Arif BM (2005) Gene organization and sequencing of the Choristoneura fumiferana defective nucleopolyhedrovirus genome. J Gen Virol 86: 945-961.

13. Thumbi DK, Beliveau C, Cusson M, Lapointe R, Lucarotti CJ (2013) Comparative Genome Sequence Analysis of Choristoneura occidentalis Freeman and C. rosaceana Harris (Lepidoptera: Tortricidae) Alphabaculoviruses. PLoS One 8: e68968.

14. Hyink O, Dellow RA, Olsen MJ, Caradoc-Davies KM, Drake K, et al. (2002) Whole genome analysis of the Epiphyas postvittana nucleopolyhedrovirus. J Gen Virol 83: 957-971.

15. Ikeda M, Shikata M, Shirata N, Chaeychomsri S, Kobayashi M (2006) Gene organization and complete sequence of the Hyphantria cunea nucleopolyhedrovirus genome. J Gen Virol 87: 2549-2562.

16. Chen YR, Wu CY, Lee ST, Wu YJ, Lo CF, et al. (2008) Genomic and host range studies of Maruca vitrata nucleopolyhedrovirus. J Gen Virol 89: 2315-2330.

17. Ahrens CH, Russell RL, Funk CJ, Evans JT, Harwood SH, et al. (1997) The sequence of the Orgyia pseudotsugata multinucleocapsid nuclear polyhedrosis virus genome. Virology 229: 381-399.

18. Qian H, Zhang Y, Wu Y, Sun P, Zhu S, et al. (2013) Analysis of the genomic sequence of Philosamia cynthia nucleopolyhedrin virus and comparison with Antheraea pernyi nucleopolyhedrin virus. BMC Genomics 14: 115.

19. Harrison RL, Lynn DE (2007) Genomic sequence analysis of a nucleopolyhedrovirus isolated from the diamondback moth, Plutella xylostella. Virus Genes 35: 857-873.

20. Harrison RL, Bonning BC (2003) Comparative analysis of the genomes of Rachiplusia ou and Autographa californica multiple nucleopolyhedroviruses. J Gen Virol 84: 1827-1842.

21. Wang YS, Huang GH, Cheng XH, Wang X, Garretson TA, et al. (2012) Genome of Thysanoplusia orichalcea multiple nucleopolyhedrovirus lacks the superoxide dismutase gene. J Virol 86: 11948-11949.

22. Nakai M, Goto C, Kang W, Shikata M, Luque T, et al. (2003) Genome sequence and organization of a nucleopolyhedrovirus isolated from the smaller tea tortrix, Adoxophyes honmai. Virology 316: 171-183.

23. Hilton S, Winstanley D (2008) Genomic sequence and biological characterization of a nucleopolyhedrovirus isolated from the summer fruit tortrix, Adoxophyes orana. J Gen Virol 89: 2898-2908.

24. Harrison RL (2009) Genomic sequence analysis of the Illinois strain of the Agrotis ipsilon multiple nucleopolyhedrovirus. Virus Genes 38: 155-170.

25. Jakubowska AK, Peters SA, Ziemnicka J, Vlak JM, van Oers MM (2006) Genome sequence of an enhancin gene-rich nucleopolyhedrovirus (NPV) from Agrotis segetum: collinearity with Spodoptera exigua multiple NPV. J Gen Virol 87: 537-551.

26. van Oers MM, Abma-Henkens MH, Herniou EA, de Groot JC, Peters S, et al. (2005) Genome sequence of Chrysodeixis chalcites nucleopolyhedrovirus, a baculovirus with two DNA photolyase genes. J Gen Virol 86: 2069-2080.

27. Zhu SY, Yi JP, Shen WD, Wang LQ, He HG, et al. (2009) Genomic sequence, organization and characteristics of a new nucleopolyhedrovirus isolated from Clanis bilineata larva. BMC Genomics 10: 91.

28. Ma XC, Shang JY, Yang ZN, Bao YY, Xiao Q, et al. (2007) Genome sequence and organization of a nucleopolyhedrovirus that infects the tea looper caterpillar, Ectropis obliqua. Virology 360: 235-246.

29. Tang XD, Xiao Q, Ma XC, Zhu ZR, Zhang CX (2009) Morphology and genome of Euproctis pseudoconspersa nucleopolyhedrovirus. Virus Genes 38: 495-506.

30. Chen X, WF IJ, Tarchini R, Sun X, Sandbrink H, et al. (2001) The sequence of the Helicoverpa armigera single nucleocapsid nucleopolyhedrovirus genome. J Gen Virol 82: 241-257.

31. Zhang CX, Ma XC, Guo ZJ (2005) Comparison of the complete genome sequence between C1 and G4 isolates of the Helicoverpa armigera single nucleocapsid nucleopolyhedrovirus. Virology 333: 190-199.

32. Ogembo JG, Caoili BL, Shikata M, Chaeychomsri S, Kobayashi M, et al. (2009) Comparative genomic sequence analysis of novel Helicoverpa armigera nucleopolyhedrovirus (NPV) isolated from Kenya and three other previously sequenced Helicoverpa spp. NPVs. Virus Genes 39: 261-272.

33. Tang P, Zhang H, Li Y, Han B, Wang G, et al. (2012) Genomic sequencing and analyses of HearMNPV--a new Multinucleocapsid nucleopolyhedrovirus isolated from Helicoverpa armigera. Virol J 9: 168.

34. Chen X, Zhang WJ, Wong J, Chun G, Lu A, et al. (2002) Comparative analysis of the complete genome sequences of Helicoverpa zea and Helicoverpa armigera single-nucleocapsid nucleopolyhedroviruses. J Gen Virol 83: 673-684.

35. Rohrmann GF, Erlandson MA, Theilmann DA (2013) The genome of a baculovirus isolated from Hemileuca sp. encodes a serpin ortholog. Virus Genes.

36. Xiao H, Qi Y (2007) Genome sequence of Leucania seperata nucleopolyhedrovirus. Virus Genes 35: 845-856.

37. Kuzio J, Pearson MN, Harwood SH, Funk CJ, Evans JT, et al. (1999) Sequence and analysis of the genome of a baculovirus pathogenic for Lymantria dispar. Virology 253: 17-34.

38. Nai YS, Wu CY, Wang TC, Chen YR, Lau WH, et al. (2010) Genomic sequencing and analyses of Lymantria xylina multiple nucleopolyhedrovirus. BMC Genomics 11: 116.

39. Choi JB, Heo WI, Shin TY, Bae SM, Kim WJ, et al. (2013) Complete genomic sequences and comparative analysis of Mamestra brassicae nucleopolyhedrovirus isolated in Korea. Virus Genes 47: 133-151.

40. Li Q, Donly C, Li L, Willis LG, Theilmann DA, et al. (2002) Sequence and organization of the Mamestra configurata nucleopolyhedrovirus genome. Virology 294: 106-121.

41. Li L, Li Q, Willis LG, Erlandson M, Theilmann DA, et al. (2005) Complete comparative genomic analysis of two field isolates of Mamestra configurata nucleopolyhedrovirus-A. J Gen Virol 86: 91-105.

42. Li L, Donly C, Li Q, Willis LG, Keddie BA, et al. (2002) Identification and genomic analysis of a second species of nucleopolyhedrovirus isolated from Mamestra configurata. Virology 297: 226-244.

43. Thumbi DK, Eveleigh RJ, Lucarotti CJ, Lapointe R, Graham RI, et al. (2011) Complete sequence, analysis and organization of the Orgyia leucostigma nucleopolyhedrovirus genome. Viruses 3: 2301-2327.

44. WF IJ, van Strien EA, Heldens JG, Broer R, Zuidema D, et al. (1999) Sequence and organization of the Spodoptera exigua multicapsid nucleopolyhedrovirus genome. J Gen Virol 80 ( Pt 12): 3289-3304.

45. Harrison RL, Puttler B, Popham HJ (2008) Genomic sequence analysis of a fast-killing isolate of Spodoptera frugiperda multiple nucleopolyhedrovirus. J Gen Virol 89: 775-790.

46. Wolff JL, Valicente FH, Martins R, Oliveira JV, Zanotto PM (2008) Analysis of the genome of Spodoptera frugiperda nucleopolyhedrovirus (SfMNPV-19) and of the high genomic heterogeneity in group II nucleopolyhedroviruses. J Gen Virol 89: 1202-1211.

47. Simon O, Palma L, Beperet I, Munoz D, Lopez-Ferber M, et al. (2011) Sequence comparison between three geographically distinct Spodoptera frugiperda multiple nucleopolyhedrovirus isolates: Detecting positively selected genes. J Invertebr Pathol 107: 33-42.

48. Simon O, Palma L, Williams T, Lopez-Ferber M, Caballero P (2012) Analysis of a naturally-occurring deletion mutant of Spodoptera frugiperda multiple nucleopolyhedrovirus reveals sf58 as a new per os infectivity factor of lepidopteran-infecting baculoviruses. J Invertebr Pathol 109: 117-126.

49. Pang Y, Yu J, Wang L, Hu X, Bao W, et al. (2001) Sequence analysis of the Spodoptera litura multicapsid nucleopolyhedrovirus genome. Virology 287: 391-404.

50. Breitenbach JE, El-Sheikh el SA, Harrison RL, Rowley DL, Sparks ME, et al. (2013) Determination and analysis of the genome sequence of Spodoptera littoralis multiple nucleopolyhedrovirus. Virus Res 171: 194-208.

51. Willis LG, Seipp R, Stewart TM, Erlandson MA, Theilmann DA (2005) Sequence analysis of the complete genome of Trichoplusia ni single nucleopolyhedrovirus and the identification of a baculoviral photolyase gene. Virology 338: 209-226.

52. Wormleaton S, Kuzio J, Winstanley D (2003) The complete sequence of the Adoxophyes orana granulovirus genome. Virology 311: 350-365.

53. Escasa SR, Lauzon HA, Mathur AC, Krell PJ, Arif BM (2006) Sequence analysis of the Choristoneura occidentalis granulovirus genome. J Gen Virol 87: 1917-1933.

54. Liang Z, Zhang X, Yin X, Cao S, Xu F (2011) Genomic sequencing and analysis of Clostera anachoreta granulovirus. Arch Virol 156: 1185-1198.

55. Liang Z, Zhang X, Yin X, Song X, Shao X, et al. (2013) Comparative analysis of the genomes of Clostera anastomosis (L.) granulovirus and Clostera anachoreta granulovirus. Arch Virol 158: 2109-2114.

56. Lange M, Jehle JA (2003) The genome of the Cryptophlebia leucotreta granulovirus. Virology 317: 220-236.

57. Luque T, Finch R, Crook N, O'Reilly DR, Winstanley D (2001) The complete sequence of the Cydia pomonella granulovirus genome. J Gen Virol 82: 2531-2547.

58. Ferrelli ML, Salvador R, Biedma ME, Berretta MF, Haase S, et al. (2012) Genome of Epinotia aporema granulovirus (EpapGV), a polyorganotropic fast killing betabaculovirus with a novel thymidylate kinase gene. BMC Genomics 13: 548.

59. Harrison RL, Popham HJ (2008) Genomic sequence analysis of a granulovirus isolated from the Old World bollworm, Helicoverpa armigera. Virus Genes 36: 565-581.

60. Taha A, Nour-El-Din A, Croizier L, Ferber ML, Croizier G (2000) Comparative analysis of the granulin regions of the Phthorimaea operculella and Spodoptera littoralis granuloviruses. Virus Genes 21: 147-155.

61. Zhang BQ, Cheng RL, Wang XF, Zhang CX (2012) The Genome of Pieris rapae Granulovirus. J Virol 86: 9544.

62. Hashimoto Y, Hayakawa T, Ueno Y, Fujita T, Sano Y, et al. (2000) Sequence analysis of the Plutella xylostella granulovirus genome. Virology 275: 358-372.

63. Wang Y, Choi JY, Roh JY, Liu Q, Tao XY, et al. (2011) Genomic sequence analysis of granulovirus isolated from the tobacco cutworm, Spodoptera litura. PLoS One 6: e28163.

64. Hayakawa T, Ko R, Okano K, Seong SI, Goto C, et al. (1999) Sequence analysis of the Xestia c-nigrum granulovirus genome. Virology 262: 277-297.

65. Duffy SP, Young AM, Morin B, Lucarotti CJ, Koop BF, et al. (2006) Sequence analysis and organization of the Neodiprion abietis nucleopolyhedrovirus genome. J Virol 80: 6952-6963.

66. Lauzon HA, Lucarotti CJ, Krell PJ, Feng Q, Retnakaran A, et al. (2004) Sequence and organization of the Neodiprion lecontei nucleopolyhedrovirus genome. J Virol 78: 7023-7035.

67. Garcia-Maruniak A, Maruniak JE, Zanotto PM, Doumbouya AE, Liu JC, et al. (2004) Sequence analysis of the genome of the Neodiprion sertifer nucleopolyhedrovirus. J Virol 78: 7036-7051.

68. Afonso CL, Tulman ER, Lu Z, Balinsky CA, Moser BA, et al. (2001) Genome sequence of a baculovirus pathogenic for Culex nigripalpus. J Virol 75: 11157-11165.
