# Supplementary material for: Genome Sequence and Analysis of Buzura suppressaria Nucleopolyhedrovirus: A Group II Alphabaculovirus
Source: PLoS One. 2014 Jan 24;9(1):e86450. doi: 10.1371/journal.pone.0086450 (PMC3901692; doi:10.1371/journal.pone.0086450)
Supplement: Table S2 — The ORF positions in the genomeof BusuNPV. E or L means early or late promoter motif and ORF directionrepresented by+ or –.* stands for stain HearNPV G4. a, position of granulin in CpGV genome. b, BJDP stands for DnaJ domain protein. c, PKIP stands forProtein kinase interacting. (DOCX) [file pone.0086450.s002.docx]

| name | Prom. motif | ORF | start | end | lenth (aa)  **Table S2. The ORF positions in the genome of BusuNPV.^#^** | Str. | ORF position | | | | | | | amino acid identity | | | | | | |
| --- | --- | --- | --- | --- | --- | --- | --- | --- | --- | --- | --- | --- | --- | --- | --- | --- | --- | --- | --- | --- |
|  |  |  |  |  |  |  | Ac MNPV | Hear NPV* | Ld MNPV | Se NPV | Cp GV | nele NPV | Cuni NPV | AC NPV | Hear NPV* | Ld NPV | Se NPV | Cp GV | Nele NPV | Cuni NPV |
| Polyhedrin | E,L | 1 | 1 | 741 | 246 | + | 8 | 1 | 1 | 1 | 1^a^ | 1 |  | 90% | 91% | 83% | 92% | 57% | 48% |  |
| P78/83 | L | 2 | 788 | 2419 | 543 | - | 9 | 2 | 2 | 2 | 2 |  |  | 23% | 29% | 28% | 38% | 22% |  |  |
| PK-1 | L | 3 | 2412 | 3221 | 269 | + | 10 | 3 | 3 | 3 | 3 |  |  | 38% | 46% | 48% | 51% | 35% |  |  |
| Hoar | E,L | 4 | 3315 | 5435 | 706 | - |  | 4 |  | 4 |  |  |  |  | 14% |  | 14% |  |  |  |
| ORF-5 | E | 5 | 5816 | 6733 | 305 | + |  |  |  |  |  |  |  |  |  |  |  |  |  |  |
| ODV-E56/PIF-5 | L | 6 | 6923 | 8008 | 361 | + | 148 | 15 | 14 | 6 | 18 | 23 | 102 | 56% | 53% | 60% | 55% | 46% | 36% | 17% |
| P10 | L | 7 | 8136 | 8420 | 94 | - | 137 | 21 | 41 | 130 |  |  |  | 20% | 48% | 55% | 52% |  |  |  |
| P26 | L | 8 | 8456 | 9247 | 263 | - | 136 | 22 | 40 | 129 |  |  |  | 33% | 43% | 26% | 47% |  |  |  |
| ORF-9 |  | 9 | 9379 | 9621 | 80 | + | 29 | 23 | 39 | 128 | 19 |  |  | 28% | 46% | 34% | 39% | 23% |  |  |
| LEF-6 | L | 10 | 9708 | 10181 | 157 | - | 28 | 24 | 38 | 127 | 80 |  |  | 26% | 30% | 24% | 26% | 17% |  |  |
| DBP-1 |  | 11 | 10174 | 10986 | 270 | - | 25 | 25 | 37 | 126 |  |  |  | 22% | 28% | 21% | 29% |  |  |  |
| ORF-12 | E | 12 | 11128 | 11781 | 217 | - |  |  |  |  |  |  |  |  |  |  |  |  |  |  |
| GP37 | L | 13 | 11936 | 12694 | 252 | + | 64 | 58 | 68 | 25 | 13 |  |  | 50% | 57% | 58% | 59% | 41% |  |  |
| IE-1 | E | 14 | 13374 | 14795 | 473 | - | 147 | 14 | 15 | 132 | 7 |  |  | 25% | 38% | 35% | 36% | 12% |  |  |
| ORF-15 |  | 15 | 14737 | 15153 | 138 | - |  |  |  |  |  |  |  |  |  |  |  |  |  |  |
| EP23 | L | 16 | 15141 | 15779 | 212 | + | 146 | 13 | 16 | 133 | 8 |  |  | 26% | 25% | 29% | 33% | 17% |  |  |
| ORF-17 | L | 17 | 15892 | 16170 | 92 | - | 145 | 12 | 17 | 134 | 9 | 64 |  | 46% | 57% | 58% | 53% | 24% |  |  |
| ODV-EC27 | L | 18 | 16203 | 17072 | 289 | - | 144 | 11 | 18 | 135 | 97 | 63 | 32 | 45% | 54% | 63% | 57% | 22% | 20% | 14% |
| ODV-E18 | L | 19 | 17241 | 17495 | 84 | - | 143 | 10 | 19 | 136 | 14 | 62 | 31 | 57% | 53% | 68% | 55% | 35% | 20% | 9% |
| 49K | L | 20 | 17528 | 18961 | 477 | - | 142 | 9 | 20 | 137 | 15 | 60 | 30 | 45% | 57% | 55% | 53% | 28% | 20% | 9% |
| IE-0 | E,L | 21 | 19046 | 19819 | 257 | - | 141 | 8 | 21 | 138 |  |  |  | 25% | 32% | 32% | 39% |  |  |  |
| ME-53 | E,L | 22 | 20114 | 21178 | 354 | + | 139 | 16 | 23 | 7 | 143 |  |  | 18% | 22% | 34% | 27% | 12% |  |  |
| P74 | E | 23 | 21271 | 23259 | 662 | + | 138 | 20 | 27 | 131 | 60 | 47 | 74 | 57% | 55% | 60% | 56% | 42% | 38% | 34% |
| cathepsin | L | 24 | 23318 | 24313 | 331 | - | 127 | 56 | 78 | 16 | 11 |  |  | 66% | 44% | 63% | 53% | 42% |  |  |
| P47 | E,L | 25 | 24511 | 25692 | 393 | + | 40 | 35 | 48 | 115 | 68 | 46 | 73 | 54% | 51% | 64% | 63% | 43% | 22% | 15% |
| DBP-2 | E | 26 | 25769 | 26731 | 320 | - | 25 | 25 | 47 | 126 | 81 | 14 |  | 26% | 27% | 33% | 27% | 16% | 14% |  |
| ADPRase | E,L | 27 | 26910 | 27617 | 235 | + | 38 | 33 | 46 | 118 | 69 |  |  | 54% | 51% | 56% | 60% | 38% |  |  |
| LEF-11 | L | 28 | 27545 | 27928 | 127 | + | 37 | 32 | 45 | 119 | 58 | 15 |  | 28% | 33% | 39% | 40% | 28% | 24% |  |
| 39K | L | 29 | 27885 | 28736 | 283 | + | 36 | 31 | 44 | 120 | 57 |  |  | 33% | 33% | 42% | 31% | 10% |  |  |
| ORF-30 |  | 30 | 28773 | 29192 | 139 | + |  |  |  |  |  |  |  |  |  |  |  |  |  |  |
| ORF-31 |  | 31 | 29284 | 29499 | 71 | - |  |  |  |  |  |  |  |  |  |  |  |  |  |  |
| ubiquitin | L | 32 | 29550 | 29786 | 78 | - |  |  |  | 123 |  |  |  |  |  |  | 82% |  |  |  |
| ORF-33 | L | 33 | 29855 | 30397 | 180 | + | 34 | 27 | 42 | 124 |  |  |  | 26% | 52% | 41% | 53% |  |  |  |
| VEF-1 |  | 34 | 30449 | 30631 | 60 | - |  |  |  |  |  |  |  |  |  |  |  |  |  |  |
| LEF-9 |  | 35 | 30721 | 32235 | 504 | - | 62 | 55 | 64 | 97 | 117 | 37 | 59 | 66% | 68% | 69% | 69% | 51% | 35% | 17% |
| FP25K | L | 36 | 32360 | 33007 | 215 | + | 61 | 53 | 63 | 98 | 118 |  |  | 50% | 55% | 22% | 68% | 30% |  |  |
| BRO-A | L | 37 | 33164 | 33598 | 144 | + |  | 60 | 32 |  |  |  |  |  | 32% | 27% |  |  |  |  |
| ChaB2 | L | 38 | 33665 | 33940 | 91 | + | 60 | 52 | 62 | 100 |  |  |  | 37% | 40% | 43% | 39% |  |  |  |
| ChaB1 | E,L | 39 | 34098 | 34622 | 174 | + | 59 | 51 | 61 | 101 |  |  |  | 32% | 33% | 29% | 28% |  |  |  |
| ORF-40 | E,L | 40 | 34681 | 35178 | 165 | - | 57 | 50 | 60 | 102 |  |  |  | 37% | 38% | 45% | 45% |  |  |  |
| ORF-41 |  | 41 | 35494 | 35868 | 124 | - | 55 | 48 | 58 | 103 |  |  |  | 29% | 46% | 45% | 16% |  |  |  |
| VP1054 |  | 42 | 36026 | 37036 | 336 | - | 54 | 47 | 57 | 105 | 138 | 83 | 8 | 44% | 47% | 52% | 54% | 27% | 19% | 14% |
| ORF-43 | L | 43 | 37087 | 37308 | 73 | + |  |  |  |  |  |  |  |  |  |  |  |  |  |  |
| ORF-44 | L | 44 | 37367 | 38443 | 358 | + |  | 44 | 55 | 107 |  |  |  |  | 19% | 16% | 20% |  |  |  |
| ORF-45 | E,L | 45 | 38515 | 38934 | 139 | - | 53 | 43 | 54 | 108 | 134 | 77 | 28 | 45% | 49% | 49% | 55% | 17% | 11% | 7% |
| ORF-46 | E | 46 | 38983 | 39513 | 176 | + | 52 | 42 | 53 | 109 |  |  |  | 19% | 26% | 36% | 24% |  |  |  |
| ORF-47 | E,L | 47 | 39679 | 39840 | 53 | - |  |  |  |  |  |  |  |  |  |  |  |  |  |  |
| IAP-1 | E,L | 48 | 39955 | 40503 | 182 | + | 27 | 103 | 139 | 110 | 17 |  |  | 17% | 23% | 18% | 22% | 29% |  |  |
| BJDP^b^ |  | 49 | 40575 | 41759 | 394 | - | 51 | 39 |  | 111 |  |  |  | 17% | 17% |  | 14% |  |  |  |
| LEF-8 |  | 50 | 41780 | 44413 | 877 | + | 50 | 38 | 51 | 112 | 131 | 78 | 26 | 49% | 54% | 52% | 56% | 42% | 30% | 4% |
| Chitinase | L | 51 | 44647 | 46344 | 565 | + | 126 | 41 | 70 | 19 | 10 |  |  | 68% | 64% | 67% | 63% | 58% |  |  |
| P26 |  | 52 | 46505 | 47200 | 231 | - | 136 | 22 | 40 | 87 |  |  |  | 14% | 15% | 17% | 39% |  |  |  |
| ORF-53 | L | 53 | 47407 | 47643 | 78 | - |  |  | 30 |  |  |  |  |  |  | 23% |  |  |  |  |
| IAP-2 | E,L | 54 | 47704 | 48636 | 310 | - | 71 | 62 | 79 | 88 |  |  |  | 28% | 39% | 38% | 31% |  |  |  |
| PIF-6 |  | 55 | 48668 | 49036 | 122 | - | 68 | 64 | 80 | 90 | 114 | 38 | 58 | 37% | 50% | 50% | 50% | 25% | 24% | 20% |
| LEF-3 |  | 56 | 49038 | 50303 | 421 | + | 67 | 65 | 81 | 91 | 113 |  |  | 22% | 22% | 29% | 31% | 9% |  |  |
| Desmoplakin | E,L | 57 | 50391 | 53105 | 904 | - | 66 | 66 | 82 | 92 | 112 | 21 | 92 | 18% | 18% | 20% | 21% | 13% | 12% | 13% |
| DNA-pol |  | 58 | 53104 | 56274 | 1056 | + | 65 | 67 | 83 | 93 | 111 | 20 | 91 | 43% | 54% | 52% | 54% | 32% | 24% | 16% |
| ORF-59 | L | 59 | 56467 | 56847 | 126 | - | 75 | 69 | 84 | 94 | 108 |  |  | 17% | 30% | 44% | 38% | 10% |  |  |
| ORF-60 | L | 60 | 56855 | 57112 | 85 | - | 76 | 70 | 85 | 95 | 107 | 41 |  | 41% | 65% | 74% | 65% | 33% | 22% |  |
| VLF-1 | L | 61 | 57254 | 58411 | 385 | - | 77 | 71 | 86 | 82 | 106 | 42 | 18 | 65% | 71% | 69% | 62% | 29% | 26% | 20% |
| ORF-62 | L | 62 | 58454 | 58774 | 106 | - | 78 | 72 | 87 | 81 | 105 | 46 | 34 | 34% | 44% | 42% | 43% | 15% | 19% | 16% |
| GP41 | L | 63 | 58854 | 60095 | 413 | - | 80 | 73 | 88 | 80 | 104 | 44 | 33 | 42% | 54% | 56% | 50% | 31% | 25% | 12% |
| ORF-64 | L | 64 | 60067 | 60903 | 278 | - | 81 | 74 | 89 | 79 | 103 | 45 | 106 | 49% | 54% | 58% | 53% | 41% | 40% | 16% |
| TLP-20 | E,L | 65 | 60641 | 61315 | 224 | - | 82 | 75 | 90 | 78 | 102 |  |  | 27% | 40% | 35% | 41% | 15% |  |  |
| VP91/p95 | L | 66 | 61284 | 63800 | 838 | + | 83 | 76 | 91 | 77 | 101 | 82 | 35 | 40% | 43% | 41% | 45% | 22% | 23% | 22% |
| VP39 | E,L | 67 | 63931 | 64935 | 334 | - | 89 | 78 | 92 | 75 | 96 | 88 | 24 | 37% | 42% | 46% | 45% | 26% | 19% | 11% |
| LEF-4 |  | 68 | 64937 | 66304 | 455 | + | 90 | 79 | 93 | 74 | 95 | 59 | 96 | 46% | 45% | 49% | 53% | 33% | 25% | 16% |
| P33 | L | 69 | 66491 | 67249 | 252 | - | 92 | 80 | 94 | 73 | 93 | 16 | 14 | 54% | 61% | 59% | 68% | 34% | 21% | 21% |
| P18 | L | 70 | 67248 | 67721 | 157 | + | 93 | 81 | 95 | 72 | 92 | 17 | 13 | 54% | 61% | 63% | 63% | 34% | 17% | 12% |
| ODV-E25 | L | 71 | 67723 | 68394 | 223 | + | 94 | 82 | 96 | 71 | 91 | 18 | 15 | 42% | 57% | 64% | 61% | 46% | 10% | 14% |
| Helicase | L | 72 | 68525 | 72256 | 1243 | - | 95 | 84 | 97 | 70 | 90 | 58 | 89 | 41% | 46% | 53% | 51% | 23% | 19% | 12% |
| ODV-E28/PIF-4 |  | 73 | 72213 | 72731 | 172 | + | 96 | 85 | 98 | 69 | 89 | 57 | 90 | 48% | 62% | 62% | 67% | 32% | 25% | 6% |
| 38K | L | 74 | 72811 | 73716 | 301 | - | 98 | 86 | 99 | 67 | 88 | 56 | 87 | 48% | 59% | 55% | 57% | 43% | 29% | 24% |
| LEF-5 |  | 75 | 73609 | 74238 | 209 | + | 99 | 87 | 100 | 66 | 87 | 55 | 88 | 45% | 53% | 56% | 54% | 36% | 26% | 11% |
| p6.9 | L | 76 | 74475 | 74705 | 76 | - | 100 | 88 | 101 | 65 | 86 | 28 | 23 | 36% | 65% | 72% | 40% | 37% | 34% | 28% |
| C42 | L | 77 | 74763 | 75881 | 372 | - | 101 | 89 | 102 | 64 | 85 | 29 | 22 | 40% | 39% | 48% | 46% | 19% | 13% | 10% |
| P12 | L | 78 | 75937 | 76278 | 113 | - | 102 | 90 | 103 | 63 | 84 |  |  | 28% | 25% | 38% | 27% | 18% |  |  |
| P45 | E,L | 79 | 76271 | 77437 | 388 | - | 103 | 91 | 104 | 62 | 83 | 31 | 55 | 43% | 50% | 55% | 56% | 36% | 14% | 4% |
| VP80 | E | 80 | 77503 | 79881 | 792 | + | 104 | 92 | 105 | 61 |  |  |  | 13% | 19% | 17% | 21% |  |  |  |
| ORF-81 | L | 81 | 79884 | 80066 | 60 | + | 110 | 93 | 106 | 60 | 53 |  |  | 25% | 47% | 46% | 49% | 23% |  |  |
| ODV-E43 | L | 82 | 80050 | 81162 | 370 | + | 109 | 94 | 107 | 59 | 55 | 67 | 69 | 49% | 56% | 58% | 50% | 32% | 19% | 11% |
| ORF-83 | L | 83 | 81240 | 81482 | 80 | + | 108 | 95 | 108 | 58 |  |  |  | 33% | 41% | 40% | 44% |  |  |  |
| Endonuclease | L | 84 | 81717 | 82031 | 104 | + |  |  |  |  |  |  |  |  |  |  |  |  |  |  |
| NRK-1 | E | 85 | 82300 | 83364 | 354 | + |  |  | 138 | 54 | 16 |  |  |  |  | 30% | 38% | 31% |  |  |
| p43 |  | 86 | 83543 | 84688 | 381 | - | 39 |  |  |  |  |  |  | 18% |  |  |  |  |  |  |
| ORF-87 | L | 87 | 84824 | 85486 | 220 | - | 106 | 101 | 140 | 53 | 107 | 32 |  | 57% | 52% | 60% | 56% | 17% | 16% |  |
| PARG | L | 88 | 85552 | 87132 | 526 | - |  | 100 | 141 | 52 |  |  |  |  |  | 21% | 18.1% | 15% |  |  |
| ORF-89 | E | 89 | 87211 | 87708 | 165 | - |  |  |  |  |  |  |  |  |  |  |  |  |  |  |
| PIF-3 | L | 90 | 87695 | 88324 | 209 | - | 115 | 98 | 143 | 50 | 35 | 66 | 46 | 46% | 47% | 51% | 50% | 35% | 31% | 33% |
| ORF-91 | E | 91 | 88356 | 88745 | 130 | - |  |  |  |  |  |  |  |  |  |  |  |  |  |  |
| SOD | E,L | 92 | 88838 | 89332 | 164 | + | 31 | 106 | 145 | 48 | 59 |  |  | 68% | 67% | 68% | 65% | 56% |  |  |
| IAP-3 | L | 93 | 89378 | 90208 | 276 | + |  | 103 | 139 | 110 | 17 |  |  |  | 37% | 30% | 44% | 54% |  |  |
| BRO-B | L | 94 | 90315 | 90431 | 38 | - |  | 60 | 33 |  |  |  |  |  | 71% | 90% |  |  |  |  |
| BRO-C | L | 95 | 90490 | 90867 | 125 | - |  | 60 | 32 |  |  |  |  |  | 55% | 60% |  |  |  |  |
| Ctl-1 | L | 96 | 91116 | 91277 | 53 | - | 3 |  | 66 |  |  |  |  | 81% |  | 30% |  |  |  |  |
| ORF-97 |  | 97 | 91272 | 91658 | 128 | + | 117 | 110 |  | 47 |  |  |  | 32% | 41% |  | 42% |  |  |  |
| CALYX/PEP | L | 98 | 91690 | 92613 | 307 | - | 131 | 120 | 136 | 46 | 22 |  |  | 24% | 38% | 50% | 50% | 19% |  |  |
| ORF-99 | E,L | 99 | 92751 | 93893 | 380 | + |  |  |  |  |  |  |  |  |  |  |  |  |  |  |
| ORF-100 | L | 100 | 93982 | 95580 | 532 | + |  |  |  |  |  |  |  |  |  |  |  |  |  |  |
| PIF-2 | L | 101 | 95620 | 96771 | 383 | - | 22 | 132 | 119 | 35 | 48 | 52 | 38 | 64% | 71% | 65% | 71% | 52% | 43% | 49% |
| ORF-102 | E | 102 | 96845 | 97171 | 108 | + |  |  | 111 | 33 |  |  |  |  |  | 22% | 27% |  |  |  |
| PKIP^c^ | E,L | 103 | 97202 | 97708 | 168 | - | 24 | 130 | 110 | 32 |  |  |  | 16% | 33% | 29% | 35% |  |  |  |
| LEF-2 |  | 104 | 97806 | 98438 | 210 | - | 6 | 117 | 137 | 12 | 41 | 54 | 25 | 40% | 45% | 40% | 41% | 20% | 21% | 14% |
| ORF-105 | L | 105 | 98380 | 98712 | 110 | - |  |  |  | 11 |  |  |  |  |  |  | 18% |  |  |  |
| P24 | E,L | 106 | 98839 | 99513 | 224 | + | 129 | 118 | 123-125 | 10 | 71 |  |  | 41% | 57% | 47% | 57% | 22% |  |  |
| ORF-107 | E,L | 107 | 99620 | 100003 | 127 | - |  | 68 | 135 |  |  |  |  |  | 22% | 20% |  |  |  |  |
| GP16 | L | 108 | 100098 | 100394 | 98 | + | 130 | 119 | 119 | 9 |  |  |  | 38% | 25% | 10% | 40% |  |  |  |
| ORF-109 | L | 109 | 100485 | 100952 | 155 | - |  |  |  |  |  |  |  |  |  |  |  |  |  |  |
| ORF-110 |  | 110 | 100975 | 101142 | 55 | + |  |  |  |  |  |  |  |  |  |  |  |  |  |  |
| PIF-1 | L | 111 | 101552 | 103144 | 530 | + | 119 | 111 | 155 | 36 | 75 | 76 | 29 | 51% | 49% | 46% | 44% | 34% | 31% | 27% |
| ORF-112 | E | 112 | 103426 | 103629 | 67 | - | 111 | 116 | 76 |  |  |  |  | 49% | 24% | 27% |  |  |  |  |
| ORF-113 | L | 113 | 103840 | 104076 | 78 | + |  |  |  | 37 |  |  |  |  |  |  | 29% |  |  |  |
| FGF | E | 114 | 104163 | 105221 | 352 | - | 32 | 113 | 156 | 38 | 123 |  |  | 23% | 18% | 25% | 24% | 7% |  |  |
| ORF-115 | E | 115 | 105396 | 106193 | 265 | + |  |  |  | 40 |  |  |  |  |  |  | 28% |  |  |  |
| ALK-EXO | E | 116 | 106230 | 107429 | 399 | - | 133 | 114 | 157 | 41 | 125 | 33 | 54 | 41% | 43% | 43% | 44% | 33% | 19% | 18% |
| ORF-117 | L | 117 | 107466 | 108617 | 383 | - | 18 |  | 158 | 43 |  |  |  | 22% |  | 16% | 40% |  |  |  |
| ORF-118 | L | 118 | 108619 | 108987 | 122 | + | 19 | 115 | 159 | 42 |  |  |  | 24% | 25% | 29% | 40% |  |  |  |
| ORF-119 | E,L | 119 | 109036 | 109296 | 86 | - |  |  |  |  |  |  |  |  |  |  |  |  |  |  |
| F Protein | L | 120 | 109670 | 111679 | 669 | - | 23 | 133 | 130 | 8 | 31 |  | 104 | 15% | 38% | 45% | 37% | 24% |  | 14% |
| ORF-121 | E | 121 | 111948 | 114665 | 905 | + |  | 129 | 129 | 30 |  |  |  |  | 26% | 32% | 28% |  |  |  |
| ORF-122 |  | 122 | 114752 | 115429 | 225 | - | 17 | 128 | 128 | 29 |  |  |  | 24% | 29% | 24% | 26% |  |  |  |
| ORF-123 | E | 123 | 115462 | 116001 | 179 | - |  |  | 127 | 28 |  |  |  |  |  | 26% | 25% |  |  |  |
| EGT | E | 124 | 116194 | 117735 | 513 | - | 15 | 126 | 125 | 27 | 141 |  |  | 45% | 52% | 49% | 59% | 37% |  |  |
| ORF-125 | L | 125 | 117860 | 118201 | 113 | - |  |  | 124 | 15 |  |  |  |  |  | 29% | 32% |  |  |  |
| LEF-1 |  | 126 | 118263 | 118946 | 227 | + | 14 | 124 | 123 | 14 | 74 | 65 | 45 | 39% | 46% | 46% | 48% | 36% | 30% | 20% |
| 38.7K |  | 127 | 118953 | 120053 | 366 | + | 13 | 123 | 122 | 13 | 73 |  |  | 21% | 34% | 25% | 42% | 15% |  |  |

**#** E or L means early or late promoter motif and ORF direction represented by + or –. * stands for stain HearNPV G4. a Position of granulin in CpGV genome. b BJDP stands for DnaJ domain protein. c PKIP stands for Protein kinase interacting.
